# From tokenism to meaningful engagement: best practices in patient involvement in an EU project

**DOI:** 10.1186/s40900-015-0004-9

**Published:** 2015-06-25

**Authors:** David Supple, Amanda Roberts, Val Hudson, Sarah Masefield, Neil Fitch, Malayka Rahmen, Breda Flood, Willem de Boer, Pippa Powell, Scott Wagers, on behalf of the U-BIOPRED PIP group

**Affiliations:** 1 UBIOPRED Patient Input Platform, ELF, Sheffield, UK; 2 European Lung Foundation (ELF), Sheffield, UK; 3 Biosci Consulting, Maasmechelen, Belgium; 4 Asthma UK, London, UK; 5 European Federation of Asthma and Airways Diseases Patients’ Associations (EFA), Brussels, Belgium; 6 Lung Foundation (Longfonds), Amersfoort, Netherlands

**Keywords:** EU project, Patient involvement, Research, Severe asthma

## Abstract

This commentary talks about patient involvement in one of the biggest EU projects to date—U-BIOPRED. It describes how people and carers of people with asthma have been able to develop and drive their input and have their voice heard among the >200 healthcare professional project members.

Five key principles for the success of the patient involvement group are presented: involve early, involve deeply, have patients feedback on project progress, include patients in dissemination and help patients convey their own story.

This group has been used as an example for other EU-funded projects, and the patient involvement group will be maintained after the end of the project to ensure that their experience and knowledge can help develop best practice in the future.

## Background

The concept of patient and patient representative (e.g. people living with a condition, carers, patient organisations), which will be referred to as patient throughout the paper, involvement in healthcare and research was introduced more than 20 years ago [1, 2]. After a long journey, patient involvement in research is now being widely accepted as important. The available evidence on its use and impact is slowly growing, primarily in the UK [3–6], but also internationally [7, 8]. In practice, involvement is often only ‘tokenistic’—and this is particularly true for EU-funded projects (http://www.imi.europa.eu/sites/default/files/uploads/documents/Publications/SurveyPatientsCalls1to6.pdf). In the report by the Innovative Medicines Initiative (IMI) looking at patient involvement in its first six funded projects, two thirds stated that they had patient involvement in their projects. However, the majority of patient involvement was in the form of clinical trials or other forms or research where patients provided samples. Hence, patients were used as subjects to be studied rather than having an influence on the project itself. From direct experience, the European Lung Foundation (ELF) are often asked to input into EU projects, usually with the request to help with dissemination of the findings of the study to patients and the public with little involvement in the planning and delivery of the project. One of their key recommendations from the IMI study was to “support involvement of patients in the early stages (inception phase, consortium building, scoping) to ensure patient participation is an integrated part of the project with adequate funding”.

Several attempts have been made to improve patient involvement in this arena, such as VALUE+, which aimed to exchange information, experiences and good practice regarding the meaningful involvement of patients and patient organisations in EU-supported health projects (www.eu-patient.eu/globalassets/projects/valueplus/value-toolkit.pdf).

The 2014 European Parliament’s calendar of external events began with a workshop organised by the European Federation of Allergy and Airways Disease Associations (EFA; www.efanet.org) and hosted by Catherine Stihler, MEP (17 June 2014). The main focus was to give researchers and patients the opportunity to share their visions on how innovative EU-funded research can positively impact the lives of people with lung conditions in Europe and how meaningful patient involvement in these projects can become a model for the future.

During the panel discussion, Catherine Stihler highlighted the most important next step in ensuring meaningful patient involvement—to effectively communicate the value of meaningful patient involvement in EU projects. The panel also agreed that there is a need for the publication of best practice papers in this arena for information and evidence on the impact of patient involvement. This paper is a response to that action point, and this is why it is the perfect time for *Research Involvement and Engagement* to come into being to provide a journal dedicated to this kind of publication.

### Main text: the U-BIOPRED experience

IMI was launched under the EU Framework 7 programme as a public-private partnership focused on bottlenecks in drug discovery. IMI clearly identified patients as important stakeholders. U-BIOPRED (Unbiased Biomarkers in Prediction of Respiratory Disease Outcomes) is an IMI-funded EU research project using information and samples from adults and children to learn more about different types of asthma to ensure better diagnosis and treatment for each person [9]. The project had a total funding of €26,925,702. Of this, €488,900 was for patient organisation and patient involvement in the project (2 %).

The U-BIOPRED project was one of the first IMI projects. A Patient Input Platform (PIP) was established at the start of U-BIOPRED to work together to provide the patients’ perspective, support and insight into the project’s different research and dissemination processes, with a view to helping the research stay ‘connected’ to the patient experience and needs. During the submission phase of the grant, two national (the Lung Foundation (Netherlands) and Asthma UK (UK)) and one European patient organisation (EFA), as well as the ELF and its patient organisation network, were asked to provide input into the initial proposals. Patients from these groups then went on to being part of the project. Patients were also included in the project’s multidisciplinary ethics and safety monitoring boards as well as the project’s strategic advisory board. Patients took part on the U-BIOPRED boards on a completely voluntary basis using their personal titles. Travel and lodgings were covered for each meeting, but no payment was made to the individuals for the time taken out of work and home life. The patients involved in the project were supported by their individual organisation’s secretariat for the duration of the project and centrally by a coordinator and responsible individual in the project who was recompensed for involvement through the patient organisation for which he worked.

From the outset, U-BIOPRED included patient organisations, and the PIP, not just as advisers but also as participating partners in the project. Accordingly, U-BIOPRED has strived to increase patient involvement in research beyond tokenism. The objectives of U-BIOPRED are ambitious. Some said too ambitious at the outset. However, now more than 4 years into the project, it is clear that U-BIOPRED will deliver on all of its objectives. This is in no small part due to the dedication and hard work of all project partners and the commitment to work in a highly integrated manner, more like a virtual network than a loose collection of separate centres of excellence. Patients are very much a part of this U-BIOPRED virtual network and have, in fact, aided its cohesion as they transcend work package boundaries and give a much-needed overview of the project as a whole.

As the meaningful involvement of patients has spread throughout U-BIOPRED, a number of best practices have emerged. These best practices are outlined below with examples of how they were implemented and their impact on the project as a whole.

### Involve early

Patient input is often most impactful in the project formation phase [10–14]. Researchers tend to have a focus on scientific questions and less on the wider context of disease. Patients can bring the perspective of what it is like to live with a disease, or several diseases/comorbidities and the balancing act that is required to manage them. Early patient input ranges from practical aspects, such as how to prepare documents to be understood by people without a background in science and how much a study participant should be asked to do per study visit, to more strategic input, such as how to recruit study participants from a representative breadth of socioeconomic backgrounds.

#### In practice

By having patient organisations, and their patients, as funded partners, a patient perspective was incorporated into even the earliest drafts of the project proposal and work plan. These organisations (Asthma UK and Lung Foundation) invited their patient members to join the PIP, and get involved by, for example, reviewing the informed consent forms and study protocol. As a result, changes were made to both the consent forms, practical parts of the protocols directly affecting study participants, and the structure of the study visits. For the consent forms, the main issues highlighted by the PIP were as follows: there were too many forms in different formats and hence they were harmonised and reduced in number, to ensure that the forms were available in all appropriate languages and the issue of support for completing the questions was highlighted so this could be provided on site at clinical centres. For the study visits, they were reduced in number to one and therefore more procedures were carried out in one visit. This was due to the patient perspective that taking time out of work to go to hospital was difficult, and if you were going to attend, it would be better to do in one go. The paediatric study visits were modified to better accommodate the reality of a parent having to bring the child to a study visit and the child being able to tolerate the length of the visit. The visits were therefore, in this instance, made shorter. In addition, some safety aspects of study procedures and feedback of general study results were missing from the information sheet and added after the PIP advice.

#### Impact

A more participant-friendly study design and consent form helped with recruitment and participant withdrawal. The lack of studies on paediatric patients is one of the frequently highlighted gaps in translational research [15]. U-BIOPRED has delivered a paediatric cohort of 299 participants: 102 severe asthma school age, 58 severe asthma pre-school, 83 mild/moderate asthma school age and 56 mild/moderate asthma pre-school participants. Early patient involvement in study design helped to make this possible. Often lacking from clinical studies, study participants were also informed about the study progress during the study subsequent to the intervention of the PIP and communication action by the patient organisations, such as adding a newsletter sign-up page to the website, sending twice yearly project updates to the participants, creating a Twitter page and LinkedIn group.

### Involve deeply

In order to have meaningful input, patient involvement needs to be more than a patient meeting at the beginning and the end of a project [16]. There is a perception that patients either will not understand or would not be interested in the day-to-day operations of a research project. This often means that patients are given simplified and inaccurate explanations of how a project is progressing, what the challenges are and what the results mean. In reality, the more patients are involved in the day-to-day activities of the project, and the more they are informed about its progress, the more they can understand, contribute and positively impact.

#### In practice

In U-BIOPRED, PIP members take part in the research working groups, not only the annual meetings but also the monthly or even weekly operational conference calls. One of the studies in U-BIOPRED will establish a research methodology for giving the common cold virus to people with asthma to make their asthma worse so that researchers can test the effectiveness of new therapies to treat the common cold in people with asthma. If we wait for someone to get worse, it is difficult to involve them in a coordinated study, plus getting a cold in real life is much more serious than the mild worsening provoked by the virus dose given in the study. Patient representatives regularly attend the operation calls for this study and have a deep understanding of the issues being faced, making them able to participate in the decision-making. Decisions made with patient input have included limiting the number of questionnaires, deciding to include more centres to achieve recruitment goals, decision to not have trials using animals and the need to develop information sheets on rhinovirus and bronchoscopy (final products produced with the patients telling their experiences can be found here: http://www.europeanlung.org/en/projects-and-research/projects/u-biopred/news-and-events/news/what-is-a-bronchoscopy-and-why-is-it-important-in-u-biopred).

#### Impact

Patient perception incorporated into decision-making and issue resolution means more efficiency. Instead of finding out months into the study that study participants were withdrawing from the study because of too many questionnaires, the protocol was modified in a proactive fashion thanks to patient input. The production of factsheets that answered study participants’ questions about certain aspects of the trials and procedures also helped recruitment.

### Have patients feedback of project progress

There are no better taskmasters than those who have the biggest stake in the success of a project—the people who the outcomes of the study will impact the most in the long run.

#### In practice

In the initial stages of U-BIOPRED, it was motivating to hear the patient representatives who attended the annual meeting saying that they wanted the project to “just get on with it” instead of all the intense discussions that were taking place around the project setup and resulting issues. This was also a reflection of the learning process for the patient representatives. The amount of effort and complexities in setting up an ambitious project like U-BIOPRED was a surprise to them. The patients gained real insight and experience through being party to this process. This has given them the knowledge and experience to get involved in other EU projects.

#### Impact

At a point when positivity was low within the project because of the difficulties being faced, it was helpful to have the patient voice urging the project forward, motivating the project members to work together. As project coordinator Prof. Peter Sterk stated during the 2013 U-BIOPRED annual general meeting in Barcelona—“The voice of the PIP here today encouraging us to move forward to fulfil our project goals is what we need to hear to push us forward with even more commitment and vigor in the coming year”. It forced the project partners to reflect on why they were doing this study, i.e. for the patients’ health.

### Include patients in dissemination

Patient advocates are very good at spreading the word about their disease and advances being made. They often have a more prominent presence on social media than clinicians and researchers, are committed to using it as a medium and can be relied upon to post regularly.

#### In practice

In U-BIOPRED, patients and patient organisations were utilised to disseminate important outcomes in U-BIOPRED. It is all too easy to focus solely on scientific publications. Yet, the general public is where the support for research comes from.

#### Impact

Patients can provide a highly valuable insight as reviewers of dissemination resources, such as checking messages for relevance and tone for the general public, and pushing for lay summaries of all publications coming out of the project. For publications coming out of U-BIOPRED, telephone calls were held with paper authors and members of the PIP. Members of PIP provided feedback to the authors about their papers, highlighted parts that they felt needed more emphasis from the patient perspective. In addition, together with the authors, the PIP helped to draft the lay summaries for each paper. PIP members have also been speakers in mainstream symposia and educational workshops at the European Respiratory Society (ERS) International Congress to speak about the project from their viewpoint.

The patients involved in the U-BIOPRED project also provided a core of social media connections that can help messages spread more widely. U-BIOPRED as a project has 170 followers on Twitter; however, regular tweets from the U-BIOPRED PIP members have a much greater reach, for example, Amanda Robert’s, Val Hudson’s and David Supple’s tweets on the project reach upwards of 4,999, 3,244 and 690 followers, respectively.

### Patients convey their own story

The story of a patient provides powerful inspiration.

#### In practice

U-BIOPRED held an ‘Asthma Art’ contest. People with asthma were asked to submit a piece of art that depicted how they felt about their asthma. The researchers and staff in U-BIOPRED then judged the submitted art. A total of 100 researchers selected 1 entry out of 12. An 18-year-old person with asthma was chosen as the winner. Her dark and claustrophobic-feeling image skillfully represents what is it like to have asthma. Patients have also contributed their experiences for information sheets on having a bronchoscopy and taking part in the cold study to support recruitment and engagement.

#### Impact

Judging those pieces of art gave the U-BIOPRED researchers the motivation to persist in moving forward despite what, at times, seemed to be insurmountable challenges. The winner of the contest has gone on to become an active patient advocate, representing herself and others with asthma. It is important and useful to remind researchers why they are doing what they are doing. There is no more powerful way to do this than when patients tell their story. In addition, on the U-BIOPRED website, there is an opportunity for people living with asthma to share their stories, and this section has been viewed 159 times and remains one of the most popular pages on the project website (http://www.europeanlung.org/en/projects-and-research/projects/u-biopred/news-and-events/art-contest-2011).

### Barriers and difficulties

Although there is a positive message about the future of patient involvement in EU-funded projects, the barriers should be noted so that action can be taken to overcome these, including:

- Language and adequate representation
  There is a necessity for patients involved in EU projects to speak English to ensure that they can understand all communications coming from the project and follow meetings. Novel ways need to be found to ensure that a wider group of patients can participate—these include multilingual surveys, focus groups being held in individual countries and resources and news being regularly translated.
- Travel capacity
  Travelling to face-to-face meetings across Europe can be difficult and time-consuming, especially for patients who are not in the best health. Issues can also arise from costs of travel insurance, etc. Where possible, involvement should be facilitated by phone and internet to ensure all those willing to participate can. The value of face-to-face meetings is still high however. And EU projects should ensure that they cover all costs that patients incur if they are able to travel to join meetings, with minimal pre-payment being required by the patient.
- Time of work or being able to get away from the home situation
  If meeting are held across Europe, there is often two or more days required to travel and take part. If these days are not recompensed, as was the case in U-BIOPRED, then it is often difficult for people with work or home commitments to take that time out. EU projects should look to following the example of organisations such as the European Medicines Agency (EMA) and consider paying a day rate.
- Duration of commitment
  The U-BIOPRED project has run for 6 years in total, and this has been a long time to commit to the project. The majority of original patients are still involved, although at some points, some have played a more active role than others. It is important to ensure that the roles people can play can be amended to fit their health situation at any time and are flexible enough to encourage commitment at whatever level is possible and of interest to them.
- Lack of knowledge on specific topics
  Sometimes, it is difficult for patients to follow the discussions and activities in EU projects due to a lack of understanding about topics such as clinical trials, procedures required to run clinical trials, terminology used to described different techniques, etc. There are many programmes and activities that have been established to try and address this issue. Two examples are EPAP (the European Ambassador Patient Ambassador Programme —www.EPAPonline.eu), which aims to provide patients with any chronic disease and carers with the essential skills and knowledge needed to interact with healthcare professionals, policymakers, researchers and journalists, and EUPATI (European Patients Academy on Therapeutic Innovation—http://www.patientsacademy.eu/index.php/en/), a more in-depth course on research, which provides scientifically reliable, objective, comprehensive information to patients on medicines research and development.
- Effective collaboration
  In order for patient involvement to be truly productive, the healthcare professionals and scientists involved in the project needs to embrace and accept the patient perspective—their views and opinions. There is no doubt that attitudes have changed dramatically even over the course of the U-BIOPRED project—with those skeptical about the impact of patient involvement at the beginning of the project being very satisfied with the input at the end. Publications such as this and published evidence of the impact of patient involvement is, however, still vitally needed to ensure that impact of patient involvement is fully understood and appreciated. ELF worked with the ERS recently to hold an online course for professionals outlining the advantages for them of public involvement (http://www.ers-education.org/events/courses/from-research-to-bedside-the-benefits-of-involving-patients-in-respiratory-healthcare.aspx). This kind of education and further training to students and scientists is vital if patient involvement in EU projects is going to continue to grow.

## Conclusions

The journey for patients working in EU projects has only just started, and the future is exciting. The more patients get involved, the more opportunities for impact can be realised.

The U-BIOPRED PIP does not stop here either. The expertise from the group will not be lost, as EFA, together with the ELF (www.europeanlung.org), is facilitating the further development and expansion of PIP as an independent group of patient advocates from across Europe who can contribute their knowledge and experience of EU research to ensure meaningful patient engagement in future EU-funded research. There are plans to evolve this group to allow it to provide advice to others and to act on a consultancy basis for other patient groups in other projects and other disease areas.

## Abbreviations

U-BIOPRED: Unbiased Biomarkers in Prediction of Respiratory Disease Outcomes
