# Erratum to: From tokenism to meaningful engagement: best practices in patient involvement in an EU project

**DOI:** 10.1186/s40900-015-0012-9

**Published:** 2015-11-16

**Authors:** David Supple, Amanda Roberts, Val Hudson, Sarah Masefield, Neil Fitch, Malayka Rahman, Breda Flood, Willem de Boer, Pippa Powell, Scott Wagers, on behalf of the U-BIOPRED PIP group

**Affiliations:** 1 UBIOPRED Patient Input Platform, ELF, Sheffield, UK; 2 European Lung Foundation (ELF), Sheffield, UK; 3 Biosci Consulting, Maasmechelen, Belgium; 4 Asthma UK, London, UK; 5 European Federation of Asthma and Airways Diseases Patients’ Associations (EFA), Brussels, Belgium; 6 Lung Foundation (Longfonds), Amersfoort, Netherlands

Unfortunately, the original version of this article [1] contained an error. The spelling of the author Malayka Rahmen name was incorrect. The correct author name can be found below:

Malayka Rahman
